# Dynamic Pax6 expression during the neurogenic cell cycle influences proliferation and cell fate choices of retinal progenitors

**DOI:** 10.1186/1749-8104-4-32

**Published:** 2009-08-17

**Authors:** Yi-Wen Hsieh, Xian-Jie Yang

**Affiliations:** 1 Jules Stein Eye Institute and Department of Ophthalmology, Molecular Biology Institute, University of California, David Geffen School of Medicine, Stein Plaza, Los Angeles, CA 90095, USA; 2 Division of Developmental Biology, Cincinnati Children's Hospital Research Foundation, Cincinnati, OH 45229, USA

## Abstract

**Background:**

The paired homeobox protein Pax6 is essential for proliferation and pluripotency of retinal progenitors. However, temporal changes in Pax6 protein expression associated with the generation of various retinal neurons have not been characterized with regard to the cell cycle. Here, we examine the dynamic changes of Pax6 expression among chicken retinal progenitors as they progress through the neurogenic cell cycle, and determine the effects of altered Pax6 levels on retinogenesis.

**Results:**

We provide evidence that during the preneurogenic to neurogenic transition, Pax6 protein levels in proliferating progenitor cells are down-regulated. Neurogenic retinal progenitors retain a relatively low level of Pax6 protein, whereas postmitotic neurons either elevate or extinguish Pax6 expression in a cell type-specific manner. Cell imaging and cell cycle analyses show that neurogenic progenitors in the S phase of the cell cycle contain low levels of Pax6 protein, whereas a subset of progenitors exhibits divergent levels of Pax6 protein upon entering the G2 phase of the cell cycle. We also show that M phase cells contain varied levels of Pax6, and some correlate with the onset of early neuronal marker expression, forecasting cell cycle exit and cell fate commitment. Furthermore, either elevating or knocking down Pax6 attenuates cell proliferation and results in increased cell death. Reducing Pax6 decreases retinal ganglion cell genesis and enhances cone photoreceptor and amacrine interneuron production, whereas elevating Pax6 suppresses cone photoreceptor and amacrine cell fates.

**Conclusion:**

These studies demonstrate for the first time quantitative changes in Pax6 protein expression during the preneurogenic to neurogenic transition and during the neurogenic cell cycle. The results indicate that Pax6 protein levels are stringently controlled in proliferating progenitors. Maintaining a relatively low Pax6 protein level is necessary for S phase re-entry, whereas rapid accumulation or reduction of Pax6 protein during the G2/M phase of the cell cycle may be required for specific neuronal fates. These findings thus provide novel insights on the dynamic regulation of Pax6 protein among neurogenic progenitors and the temporal frame of neuronal fate determination.

## Background

The *Pax6 *gene encodes an evolutionarily conserved paired homeobox protein critically involved in eye development and retinogenesis [1-5]. In both *Drosophila *and vertebrates, ectopic expression of Pax6 induces ectopic eyes or lens [2,6,7], indicating an instructive role of Pax6 in ocular tissue determination. Genetic studies have demonstrated that the dosages of Pax6 strongly impact the formation and growth of ocular tissues. Homozygous or heterozygous loss of function Pax6 mutants have either no eye or small eye phenotypes [8-11]. Conversely, increasing dosages of the Pax6 gene also lead to microphthalmia and malformed retinas [12,13]. Furthermore, Pax6 is involved in pattern formation of the vertebrate eye. Aberrant Pax6 expression results in malformation of the anterior segment of the eye [5,14-16]. Pax6 expression in the optic vesicle is required for establishing the naso-temporal axis and the dorsal characteristics of the retina [17], whereas Pax6 expressed in the distal optic cup reciprocally suppresses Pax2 in the optic stalk to establish the optic cup/stalk boundary [18]. The complex roles of Pax6 in eye development are reflected by the existence of multiple enhancers that regulate the exquisite spatial and temporal expression of Pax6 transcripts in neural and non-neural ocular tissues [17,19-21].

Multiple Pax6 isoforms are encoded by the *Pax6 *gene in vertebrates [22-24]. The canonical Pax6 and the Pax6(5a) isoform, which contains amino acid residues encoded by an alternatively spliced exon inserted within the paired domain, are commonly found among various vertebrates [22,23,25]. The mammalian Pax6 and Pax6(5a) isoforms, and their corresponding *Drosophila *homologs encoded by the *eyeless *and *eyegone *genes, exhibit distinct biological activities. Both Pax6 and eyeless can induce ectopic eye formation in the fly wing imaginal disc [2], whereas Pax6(5a) and eyegone can elicit only cell proliferation but not neuronal differentiation [26]. Similarly, in embryonic mouse cortex, Pax6 influences both cell proliferation and neuronal differentiation, but Pax6(5a) only affects proliferation [27,28]. In mice missing the transcript Pax6(5a), lens abnormalities and iris hypoplasia are observed, but development of the neural retina remains largely normal [29], suggesting that Pax6 is able to compensate for the function of Pax6(5a) in the neural retina.

During mammalian central nervous system development, multipotent progenitors undergo a transition from the preneurogenic stage, when symmetrical divisions occur and give rise to proliferative progeny, to the neurogenic stage, during which a subset of progenitors withdraw from the cell cycle and initiate neuronal differentiation. In the cortex, reducing Pax6 expression levels affects the length of the cell cycle and the progression from symmetrical to asymmetrical division among cortical progenitors [30]. Furthermore, decreased Pax6 expression leads to depletion of basal cortical progenitor and precocious neuronal differentiation [31]. In the spinal cord, altering Pax6 levels affects the expression of the proneural gene *Ngn2 *and the timing of cell cycle exit [32].

In the vertebrate retina, multipotent progenitor cells give rise to six major types of neurons and one type of glial cell in a conserved temporal order [33-38]. At the onset of retinogenesis, the central retinal progenitors enter the neurogenic stage first, while the peripheral retina remains in a preneural state [39]. When Pax6 is conditionally ablated in the peripheral retina by using the Pax6 α cre mouse, which encodes the Pax6 P0 promoter and the α enhancer driving the expression of cre recombinase, progenitor expansion is affected, resulting in a retina reduced in size [40]. In addition, in the Pax6 α cre-mediated Pax6 conditional knockout mutant, peripheral retinal progenitors are unable to express multiple neurogenic basic helix-loop-helix (bHLH) transcriptional factors and are limited to producing only amacrine interneurons [40]. Recently, it has been reported that Pax6 α cre-mediated Pax6 deletion in the peripheral retina unmasks a suppression of the cone-rod homeobox gene *Crx *by Pax6, and more centrally localized Pax6 deletion by the Chx10-cre driver also leads to the loss of progenitor multipotency [41]. Thus, Pax6 differentially expressed by the central or peripheral retinal progenitor pools may serve distinct functions during development, but nonetheless is required for generation of multiple retinal neurons.

In the mature retina, various neuronal cell types express disparate levels of Pax6. How this highly differential Pax6 expression is achieved among specific neurons remains unclear. In this study, we have analyzed Pax6 expression at the cellular level among neurogenic progenitors in the embryonic chicken retina. We demonstrate a significant down-regulation of Pax6 protein as progenitors undergo the preneurogenic to neurogenic transition. In addition, we show that while neurogenic progenitors maintain a uniformly low Pax6 level during the S phase, a subset of retinal progenitors rapidly increase or decrease their Pax6 levels as they enter G2/M phase of the cell cycle. The divergence of Pax6 protein levels correlate with the onset of differentiation for certain neurons. Moreover, disruption of Pax6 levels among neurogenic progenitor cells leads to aberrant proliferation and production of neuronal cell types. These results reveal for the first time the dynamic expression of Pax6 during the neurogenic cell cycle and highlight the importance of Pax6 protein regulation as neural progenitors adapt to different cell fates.

## Results

### Dynamic Pax6 expression during retinogenesis

**Figure 1:**
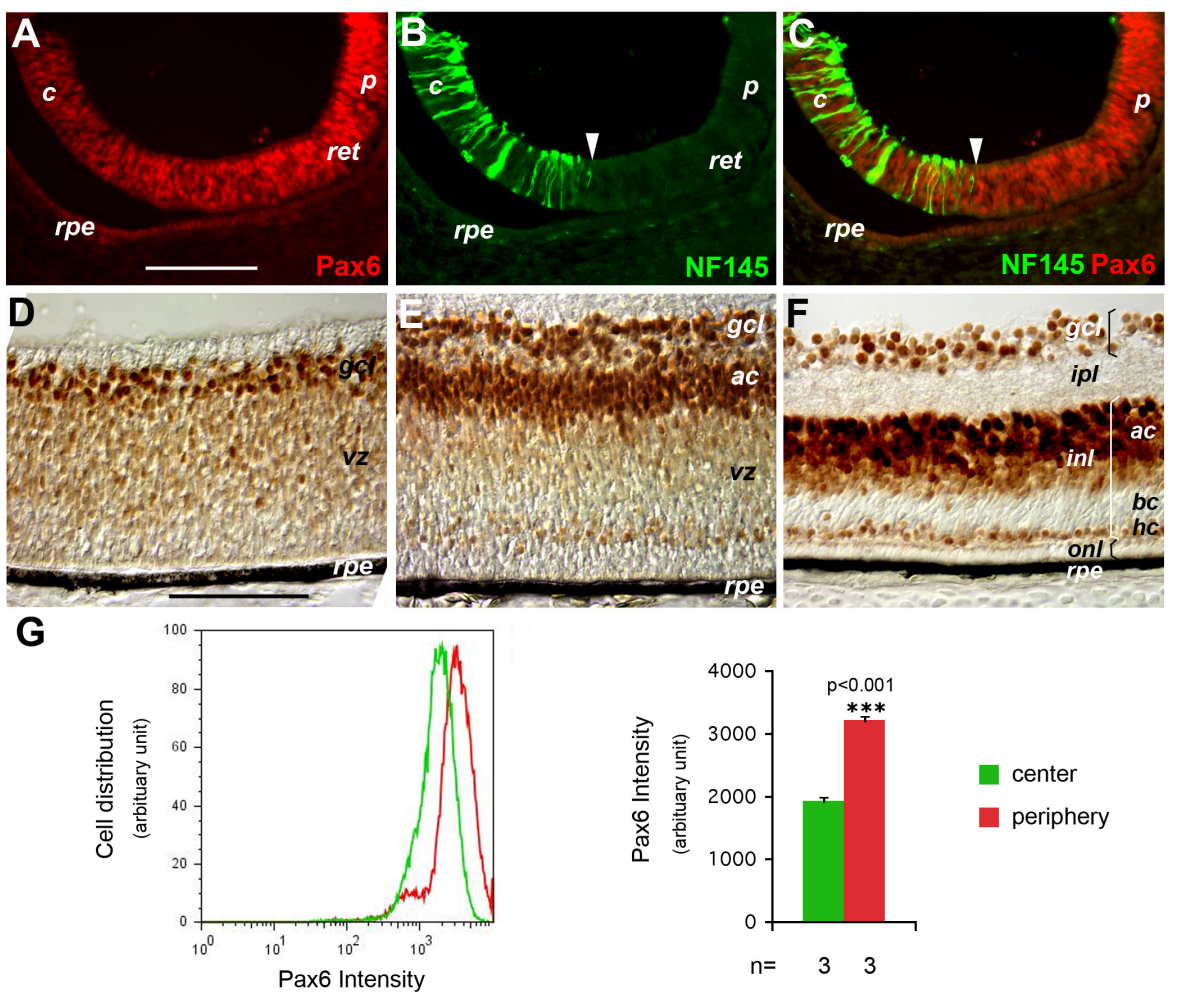
**Expression patterns of Pax6 in the developing chicken retina**. **(A-G) **Expression of Pax6 protein during the preneurogenic to neurogenic transition (A-C, G) and subsequent neurogenesis (D-F). (A-C) Immunofluorescent images of E4.5 (HH stage 24) retinas labeled for Pax6 (A), NF145 (B), and the merge of the two (C). White arrowheads in (B, C) indicate the neurogenic wave front. (D-F) Immunohistochemical images of E6.5 (HH stage 30) (D), E7 (HH stage 32) (E), and E12 (HH stage 38) (F) retinas. *ac*, amacrine cells; *bc*, bipolar cells; *c*, central retina; *gcl*, ganglion cell layer; *hc*, horizontal cells; *inl*, inner nuclear layer; *ipl*, inner plexiform layer; *onl*, outer nuclear layer; *p*, peripheral retina; *ret*, retina; *rpe*, retinal pigment epithelium; *vz*, ventricular zone. Scale bars: 100 μm; scale bar in (A) applies to (A-C), and in (D) to (D-F). (G) Quantification of Pax6 protein levels in the preneurogenic and neurogenic regions. E4.5 (HH stage 25) eyes were cut at the equator to separate the central and peripheral portions. Dissociated retinal cells from the two portions were subjected to flow cytometric analyses to quantify Pax6 protein on a per cell basis. A representative cell distribution profile with regard to Pax6 levels is shown on the left. Average Pax6 levels for the central and peripheral retinal cells (N = 3, mean ± standard error) are shown on the right. Green, central; red, peripheral. ***P < 0.001.

*Pax6 *mRNA expression in the chicken optic primordium initially begins at Hamburger and Hamilton (HH) stage 8.5 and persists at high levels throughout optic vesicle formation and optic cup morphogenesis [42]. At the onset of retinal neurogenesis at HH stage 17 [43], the expression of Pax6 protein in the central retina begins to decrease among progenitor cells. By HH stage 20, the central retina undergoing active neurogenesis showed significantly lower levels of Pax6 compared to the peripheral retina, and this change in the levels of Pax6 correlated with the propagation of the neurogenic wave, as demarcated by the emergence of NF145-positive neurons (Figure 1A–C). At the peak of retinal ganglion cell (RGC) genesis at embryonic day (E)6.5 (HH stage 30), the ventricular zone occupied by proliferative progenitor cells contained low levels of Pax6, whereas postmitotic RGCs exhibited a high level of Pax6 expression (Figure 1D). The subsequently produced amacrine cells also showed high Pax6 levels at E7 (HH stage 32; Figure 1E). By E12 (HH stage 38), when retinogenesis was near completion, amacrine cells, RGCs and horizontal cells displayed distinct levels of Pax6, in contrast to the absence of Pax6 from the photoreceptor cells and the bipolar cells (Figure 1F).

**Figure 2:**
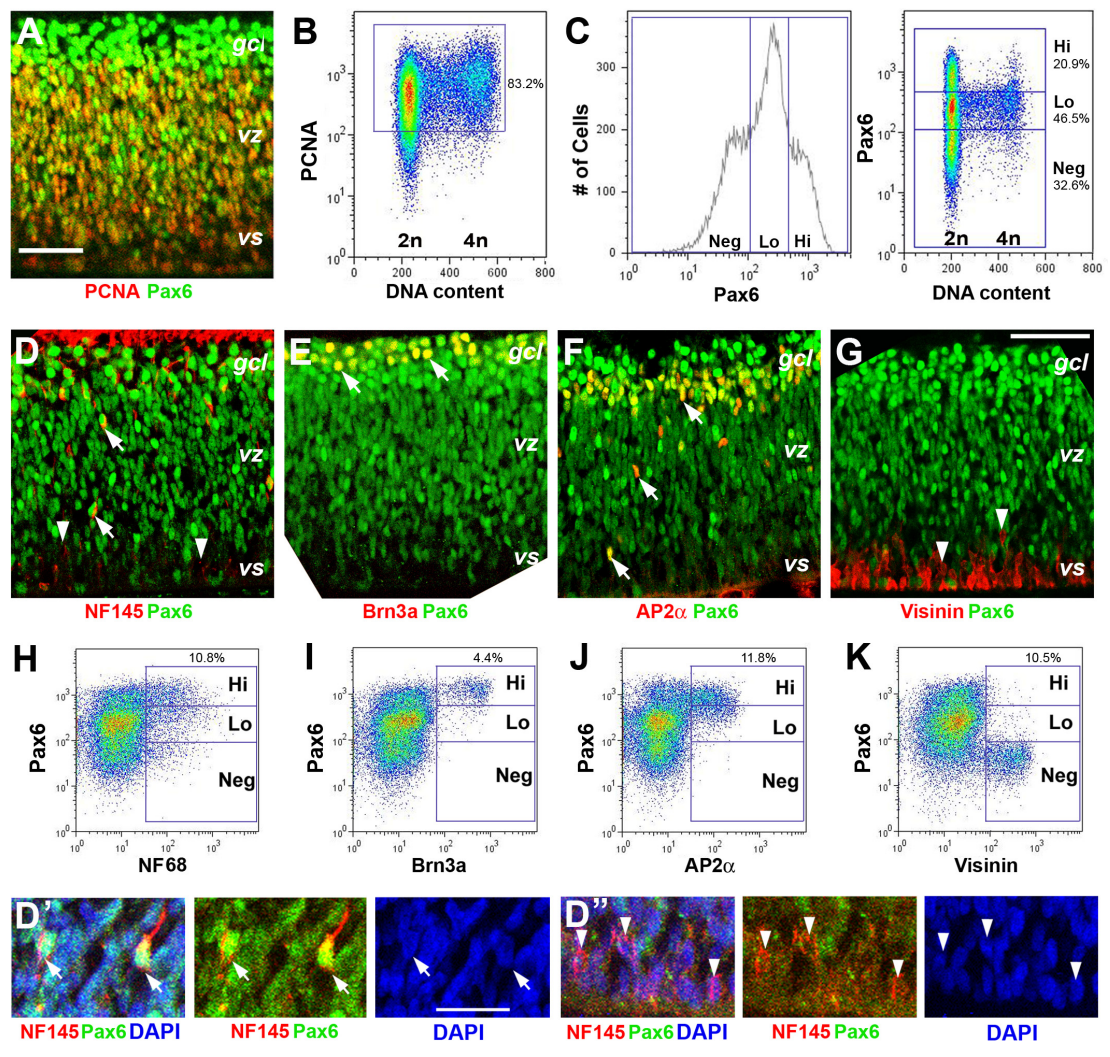
**Distinct Pax6 expression levels among progenitors and postmitotic neurons**. Immunocytochemical and flow cytometric analyses of neurogenic retinas. **(A) **Merged confocal image of PCNA (red) and Pax6 (green) co-labeled E6.5 (HH stage 30) retina. **(B) **Flow cytometry profile of E6 retinal cells based on PCNA signal and DNA content. The positions of 2n and 4n DNA contents indicate cells residing in the G1/G0 (2n) or G2/M (4n) phase. S phase cells are positioned between 2n and 4n along the DNA content axis. PCNA^+ ^cells (boxed, 83.2% of total cells) are distributed throughout the cell cycle. **(C) **Flow cytometry profiles for Pax6 levels of total cells (left panel) and Pax6 versus DNA content (right panel) at E7 (HH stage 32). Blue lines delineate Pax6^Hi ^(20.9%), Pax6^Lo ^(46.5%), and Pax6^Neg ^(32.6%) cell popuations. **(D-G) **Merged confocal images of E6.5 (HH stage 30) retinas co-labeled for Pax6 and NF145 (D), Brn3a (E), AP2α (F), or Visinin (G). **(D', D") **Higher magnification images of NF145 and Pax6 co-labeling near the ventricular surface. Arrows indicate co-labeled cells. Arrowheads point to marker-positive but Pax6-negative cells. **(H-K) **Flow cytometry profiles of Pax6 and NF68 (H), Brn3a (I), AP2α (J), and Visinin (K) at E7 (HH stage 32). Boxed regions delineate distribution of neuronal marker-positive cells according to Pax6 levels. Percentages of marker-positive cells among total cells are shown above the boxes. *gcl*, ganglion cell layer; *vs*, ventricular surface; *vz*, ventricular zone. Scale bars: 100 μm in (A, G), which applies to (D-G); 50 μm in (D'), which applies to (D', D").

To further characterize Pax6 protein levels in retinal progenitor cells and postmitotic neurons, we performed immunolabeling and flow cytometric analyses. Quantification revealed that average Pax6 signal intensity of cells residing in the peripheral halves of the retina was 1.7-fold of that found in the neurogenic central retina at HH stage 25 (Figure 1G). Quantitative measurements of Pax6 between HH stages 30 and 32 showed that the total retinal cell population existed as three distinct populations based on their Pax6 protein levels (Figure 2C, left panel). Similarly, cell distribution based on DNA content measurements also revealed three cell populations with distinct levels of Pax6 among G0/G1 cells with 2n DNA contents (Figure 2C, right panel). Based on cell density profiles of G0/G1 cells with distinct Pax6 signal levels, we delineated the boundaries between the three cell populations and designated these populations as: high Pax6 (Pax6^Hi^), low Pax6 (Pax6^Lo^), and no Pax6 (Pax6^Neg^) cells (Figure 2C). The majority of Pax6^Hi ^and Pax6^Neg ^cells had 2n DNA content and, therefore, were either G0 or G1 cells (Figure 2C; Additional file 1). The Pax6^Lo ^cells represented the largest population of cells (46.5% of total cells) at this stage (Figure 2C) and were distributed throughout the cell cycle (Figure 2C, right panel), indicating that Pax6^Lo ^cells were the proliferating progenitor cells. Immunolabeling of retinal sections showed that progenitor cell marker proliferating cell nuclear antigen (PCNA)-labeled cells were absent from the ganglion cell layer and exclusively distributed in the ventricular zone, where Pax6^Lo ^cells resided (Figure 2A). In addition, flow cytometry showed that PCNA^+ ^progenitor cells were also distributed throughout the cell cycle (Figure 2B), supporting that Pax6^Lo ^cells were progenitor cells. In contrast, Pax6^Hi ^cells were mostly co-labeled with cells positive for the neuronal markers NF145 or NF68 (Figure 2D,D',D",H) the RGC marker Brn3a (Figure 2E,I), and the amacrine cell marker AP2α (Figure 2F,J), whereas most Pax6^Neg ^cells were positively labeled with the early cone photoreceptor marker Visinin (Figure 2G,K) and a small subset was NF^+ ^(Figure 2D",H).

Together, these results demonstrate that Pax6 expression in the retina decreases as progenitor cells undergo the preneurogenic to neurogenic transition. Moreover, most neurogenic retinal progenitors maintain low levels of Pax6, whereas postmitotic neurons undergo cell type-specific elevation or reduction of Pax6.

### Cell cycle-dependent Pax6 protein expression among neurogenic progenitor cells

**Figure 3:**
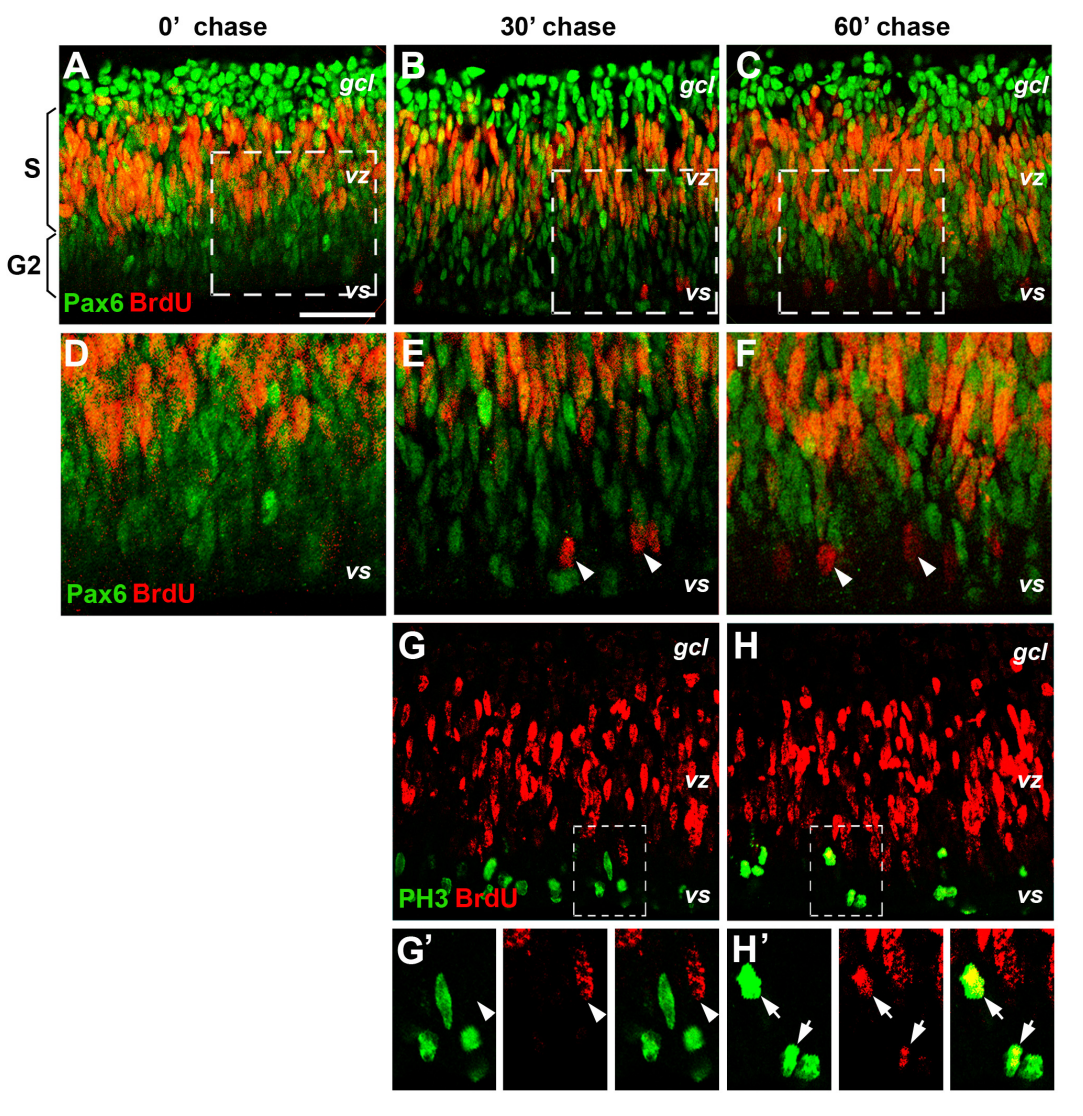
**Pax6 expression during S, G2 and M phase of the cell cycle**. Merged confocal immunofluorescent images of E6.5 (HH stage 30) retinas after a 20-minute bromodeoxyuridine (BrdU) pulse label *in vitro*. **(A-F) **Co-staining of BrdU and Pax6 at 0 (A, D), 30 (B, E), and 60 minutes (C, F) after the pulse label. The boxed regions in (A-C) are shown in (D-F), respectively, at twofold magnification. Note the appearance of BrdU^+ ^cells at the ventricular surface by the 30-minute chase time. **(G, G', H, H') **Co-staining of phospho-histone 3 (PH3) and Pax6 at 30 (G, G') or 60 minutes (H, H') after the pulse label. The boxed regions in (G, H) are shown as separate panels in (G', H'), respectively, at twofold magnification. Note the appearance of BrdU^+^PH3^+ ^double-labeled cells at the ventricular surface by the 60-minute chase time. Arrows indicate BrdU and PH3 co-labeled cells. Arrowheads point to BrdU^+ ^cells. *gcl*, ganglion cell layer; *vs*, ventricular surface; *vz*, ventricular zone. Scale bar: 100 μm; the scale bar in (A) applies to (A-C, G, H).

To examine when Pax6 levels change to result in the distinct Pax6 levels detected among postmitotic neurons, we monitored progression of Pax6 expression among progenitors following bromodeoxyuridine (BrdU) pulse labeling. Confocal imaging analyses showed that immediately after a 20-minute BrdU incubation, BrdU-positive cells were exclusively located within the middle stratum of the E6.5 (HH stage 30) retina, representing progenitor cells in the S phase of the cell cycle (Figure 3A). Most BrdU-labeled cells co-expressed low levels of Pax6 and none were detected in the outer one-third of the retina (Figure 3A,D). Within 30 minutes after the BrdU pulse, a few BrdU-positive cells began to appear in the ventricular surface (Figure 3B,E) and showed very low or no detectable Pax6 protein (Figure 3E). By 60 minutes after BrdU labeling, increasing numbers of BrdU-positive cells had reached the outer one-third of the retina (Figure 3C,F). Co-labeling of the M phase marker phospho-histone 3 (PH3) and BrdU was not detected at 30 minutes (Figure 3G,G') but was clearly visible at the ventricular surface 60 minutes after the BrdU pulse (Figure 3H,H'). These data indicate that the G2 phase of the cell cycle was as short as 60 minutes at this developmental stage. The detection of BrdU^+ ^and Pax6^Neg ^cells at the ventricular surface at 30 minutes indicated that a subset of Pax6^Lo ^progenitor cells had begun to down regulate Pax6 protein as early as the G2 phase of the cell cycle.

**Figure 4:**
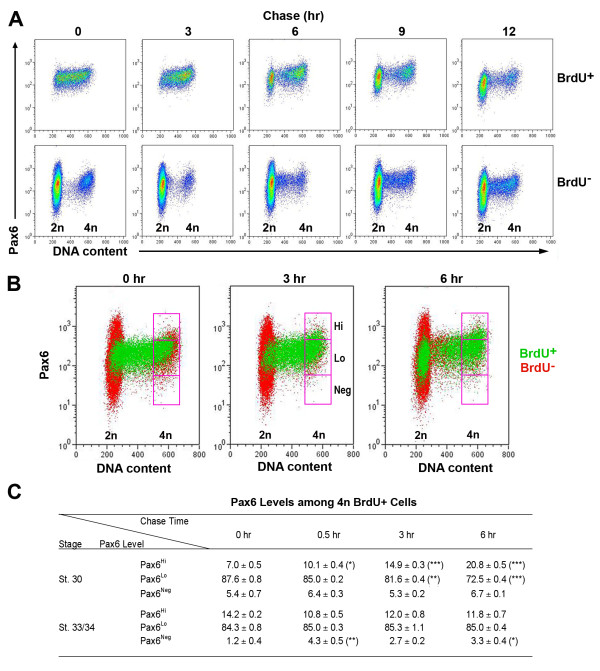
**Divergence of Pax6 protein expression in the G2/M phase of the cell cycle**. **(A) **Flow cytometric analyses of BrdU, Pax6, and DNA content among E6.5 (HH stage 30) retinal cells after a 20-minute BrdU pulse labeling. Cell density profiles of BrdU^+ ^(upper row) and BrdU^- ^(lower row) cells at various post-chase times are shown. The 0 hour point represents the time when cells are dissociated after the pulse label and then fixed and processed for flow cytometry. Each data point represents a minimum of 30,000 cells analyzed. The x-axis represents DNA content for G0/G1 (2n), S (between 2n and 4n), and G2/M (4n) phase cells. **(B) **Merged flow cytometry profiles for BrdU^+ ^(green) and BrdU^- ^(red) cells at 0, 3, and 6 hours post-BrdU pulse labeling. The purple frames subdivide G2/M phase cells (4n) according to Pax6 levels into high (Hi), low (Lo), and negative (Neg) categories. Between 0 and 6 hours of chasing, Pax6^Hi ^cells clearly increased, demonstrating the Pax6 level divergence when cells enter the G2/M phase. **(C) **Quantification of Pax6 levels at stage 30 (E6.5) and stage 33/34 (E7.5 to 8) among BrdU^+ ^cells that had entered G2/M phase (4n) as shown in the three boxed regions in (B). The percentages of 4n BrdU^+ ^cells with Pax6^Hi^, Pax6^Lo^, or Pax6^Neg ^levels among total 4n BrdU^+ ^cells at various post-chase times are shown (N = 3; mean ± standard error). Asterisks represent *P*-values between 0 hours and various chase time points: *P < 0.05; **P < 0.01; ***P < 0.001.

To confirm the confocal imaging results, we performed flow cytometry to analyze cellular Pax6 levels during S to G2/M progression. Immediately after 20-minute BrdU pulse labeling, BrdU^+ ^cells were clustered in S phase (with DNA content ranging between 2n and 4n) and showed fairly uniform levels of Pax6^Lo ^expression; whereas BrdU^- ^cells were distributed in G1/G0 phase (2n DNA content) and G2/M phase (4n DNA content) as expected (Figure 4A, 0 hour column). At 3 hours post labeling, the cohort of BrdU^+ ^cells appeared in the 4n DNA content territory (Figure 4A, 3 hour column). By 6 hours post BrdU labeling, increasing numbers of BrdU^+ ^cells appeared in G2/M phase; in addition, a fraction of the BrdU^+ ^cells clearly possessed 2n DNA content, indicating that they had completed M phase and entered G1 or G0 phase (Figure 4A, 6 hour column). Between 6 and 12 hours after pulse labeling, the BrdU^+ ^population continued to accumulate in the G1/G0 phase and decrease in the S phase, reflecting progression of the initial BrdU-labeled cohort.

We next quantified changes of Pax6 protein levels during the transition from the S phase to G2/M phase of the cell cycle. In the E6.5 (HH stage 30) retina when RGC production peaks, following a 20-minute BrdU pulse, an average of 10.7% of the cells were labeled by BrdU (Figure 4B). After a 3 or 6 hour chasing period, BrdU^+^Pax6^Hi ^cells gradually increased in the G2/M phase population (17% of total cells, with 4n DNA content) compared to the uniform Pax6^Lo ^level of the 0 hour BrdU^+ ^cells (Figure 4B). Quantification of Pax6 levels among 4n BrdU^+ ^cells showed a steady increase of BrdU^+^Pax6^Hi ^cells (Figure 4C). At 30 minutes after the pulse chase, the BrdU^+^Pax6^Hi ^cell population already showed a statistically significant increase (Figure 4C). After 6 hours of chase, the percentage of BrdU^+^Pax6^Hi ^cells had increased from 7.0% to 20.8% (Figure 4C; N = 3). Concomitantly, the Pax6^Lo ^progenitor cells decreased from 87.6% to 72.5% during the 6 hour chase period (Figure 4C; N = 3), reflecting altered Pax6 expression as progenitors progressed through the G2/M phase. However, the Pax6^Neg ^cell populations did not show statistically significant changes during the chase (Figure 4C).

**Figure 5:**
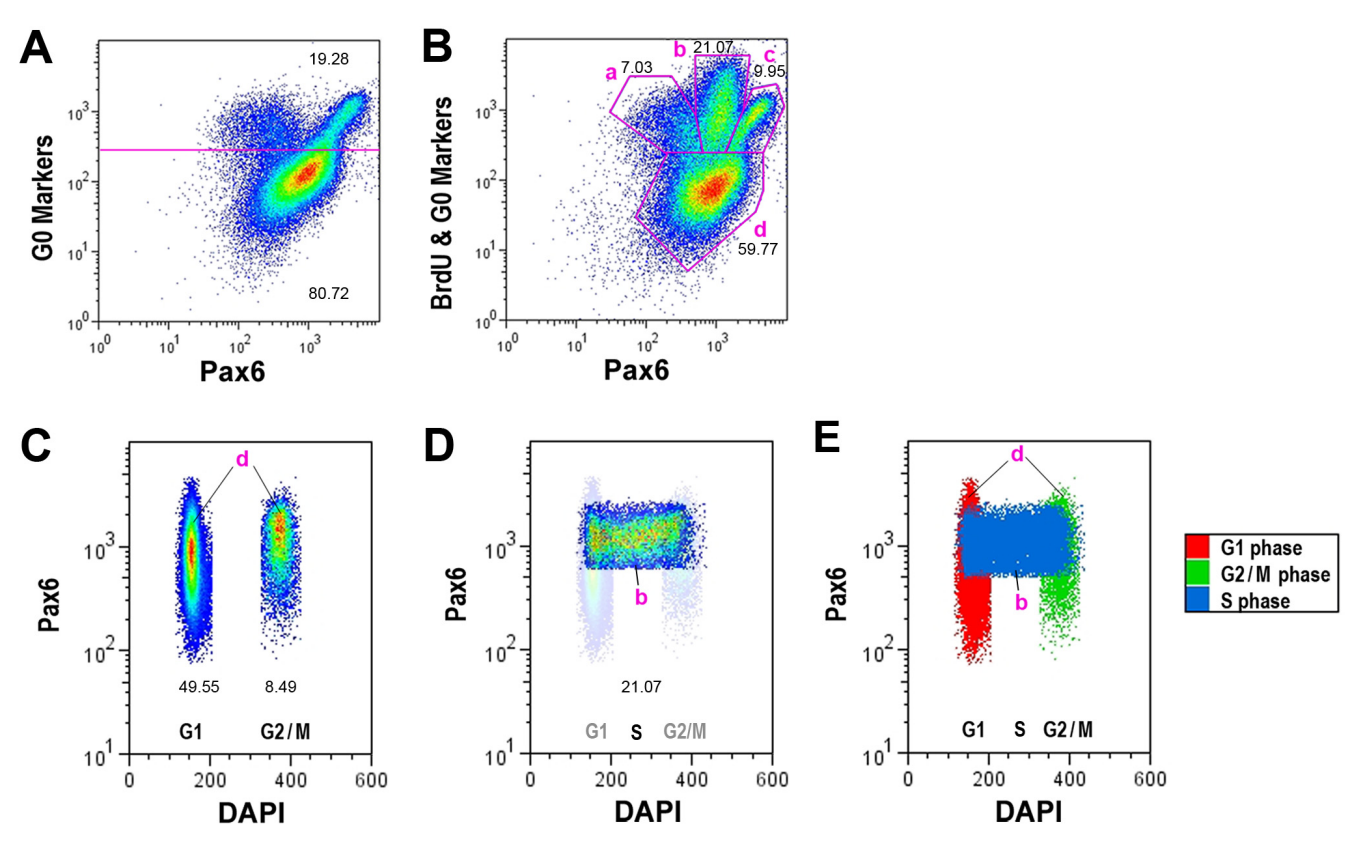
**Correlation of Pax6 levels in progenitors in different phases of the cell cycle**. Flow cytometric analyses of Pax6 levels among postmitotic cells and cycling progenitor cells at E7 (HH stage 32). **(A) **Cell density profile of total retinal cells labeled with Pax6 and a combination of neuronal markers, including Brn3a, Islet1/2, AP2a, and Visinin. Neuronal marker-positive cells (above the purple line) represent G0 cells; and neuronal marker-negative cells (below the purple line) are mostly progenitor cells. **(B) **Cell density profile of total retinal cells labeled with a combination of neuronal markers (same as in (A)), BrdU, and Pax6. Gated areas 'a' and 'c' represent postmitotic G0 phase cells; gated area 'b' represents BrdU^+ ^S phase cells; gated area 'd' consists of G1, G2, and M phase cells. **(C, D) **Cell density profiles of G1 and G2/M phase cells (C) (as gated in area 'd') and S phase cells (D) (as gated in area 'b' in (B)) according to Pax6 levels and DNA content. In (D), G1 and G2/M cells are shown in the background as a reference to S phase cells. **(E) **Merged flow cytometry profiles of G1 (red), G2/M (green), and S (blue) phase progenitor cells according to Pax6 levels and DNA content. Flow cytometry profiles shown in (A, B) were generated with >85,000 cells. Numbers within each panel indicate percentage of gated cells among total cells.

In order to monitor changes in Pax6 protein levels among progenitors during the entire cell cycle, we excluded postmitotic G0 cells by labeling with a combination of available neuronal markers, including Brn3a, Islet1/2, AP2α, and Visinin (Figure 5A). As expected, most postmitotic G0 cells positively labeled for combined neuronal markers at E7 (HH stage 32) exhibited either Pax6^Neg ^(Figure 5B, gate a) or Pax6^Hi ^profiles (Figure 5B, gate c). To identify S phase cells, we also pulse-labeled E7 cells with BrdU. Profiling of BrdU^+ ^cells showed that S phase cells contained low Pax6 levels (Figure 5B, gate b; and data not shown). We then defined the G1 and G2/M cells (Figure 5B, gate d) by exclusion of both neuronal marker-positive G0 phase cells and BrdU^+ ^S phase cells from the total cell population (Figure 5B). DNA content analyses further separated G1 and G2/M cell populations and demonstrated that G1 cells and G2/M cells exhibited a broader range of Pax6 protein levels than S phase cells (Figure 5C–E). These data again demonstrate the divergence of Pax6 levels after the S to G2/M phase transition. Furthermore, an increased Pax6^Neg ^cell population was detected in the G2/M phase, corresponding to the peak production of cone photoreceptors at E7. These results also indicate that progenitors in the G1 phase of the cell cycle show divergent Pax6 expression, which becomes more restricted upon S phase entry.

### Divergent Pax6 expression in G2/M and neuronal fate specification

**Figure 6:**
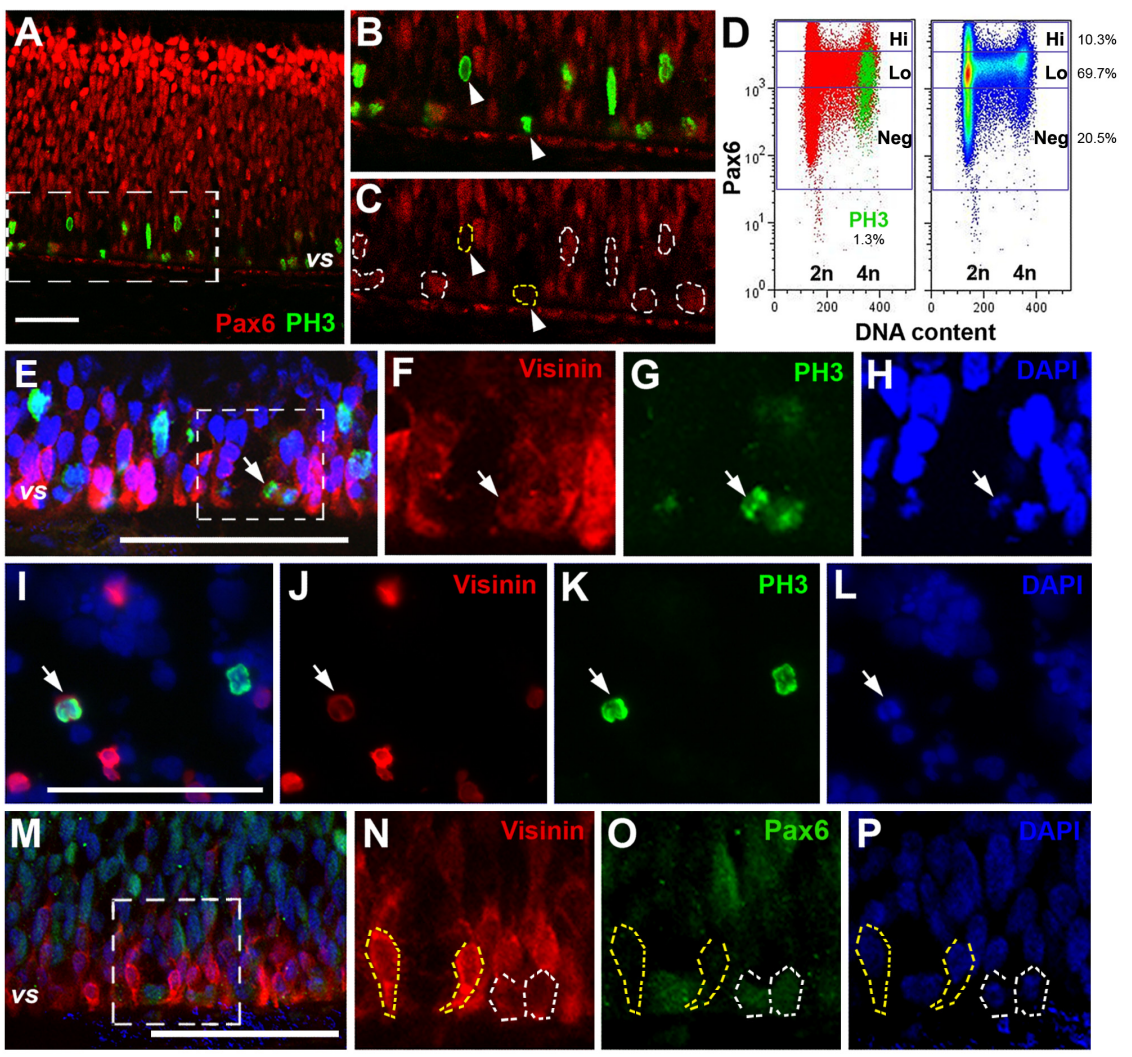
**Correlation of Pax6 changes in G2/M phase with the emergence of a neuronal marker**. **(A-C) **Confocal images of Pax6 and PH3 co-labeling at E6.5 (HH stage 30). Boxed region in (A) is shown as Pax6 and PH3 merge (B) or Pax6 alone (C) at twofold magnification, where dotted outlines in (C) encircle all PH3^+ ^cells. **(D) **Flow cytometry profiles of Pax6, PH3, and DNA content at E7 (HH stage 32). For both panels the y-axes represent Pax6 levels, while the x-axes indicate the DNA content. The left panel shows merged distribution profiles of Pax6 (red) and PH3 (green), and the percentage of PH3^+ ^cells among total cells is indicated below. The right panel shows cell density distribution with regard to Pax6 and the cell cycle. The percentages of Pax6^Hi^, Pax6^Lo^, and Pax6^Neg ^cells among total cells are indicated at the right. **(E-H) **Confocal images of Visinin, PH3, and DAPI co-staining at E6.5 (HH stage 30). The boxed region in (E) is shown as Visinin- (F), PH3- (G), or DAPI staining (H) alone at twofold magnification. **(I-L) **Co-labeling for Visinin (J), PH3 (K), DAPI (L), and merge (I) in dissociated E6 retina cells. **(M-P) **Confocal images of Visinin, Pax6, and DAPI co-staining at E6.5 (HH stage 30). The boxed region in (M) is shown as Visinin (N), Pax6 (O), or DAPI staining (P) alone at twofold magnification. Yellow dotted lines encircle Visinin^+^Pax6^Neg ^cells, whereas white dotted lines in (N-P) encircle a pair of cells at the end of the M-phase with weak Pax6 and Visinin signals. Arrows indicate co-labeled cells. Arrowheads point to marker-positive but Pax6^- ^cells. *vs*, ventricular surface. Scale bars: 100 μm.

To determine if the divergence of Pax6 levels in G2/M was correlated with progenitor cell behaviors, we performed double immunocytochemistry of Pax6 and neuronal cell markers. At E6.5 (HH stage 30) double labeling of Pax6 and the M phase marker PH3 showed that PH3^+ ^cells occupied a zone 60 μm wide from the ventricular surface (Figure 6A). Among these, a subset of PH3^+ ^cells abutting the ventricular surface showed no Pax6 expression (Figure 6B,C). Furthermore, flow cytometric analyses showed that, at E7 (HH stage 32), only a small proportion of the G2/M phase cells (16.2% of total cells) are PH3^+ ^cells (1.3% of total cells). Thus, most cells with 4n DNA content, including the majority of Pax6^Hi ^cells, were G2 phase cells (14.9% of total cells). Among PH3^+ ^M phase cells, Pax6^Hi^, Pax6^Lo ^and Pax6^Neg ^cells corresponded to 2.3%, 78.5%, and 24.5% of PH3^+ ^cells, respectively (Figure 6D).

We then examined whether G2/M progenitors with no Pax6 corresponded to committed cone photoreceptors, which at maturity contain no Pax6. Confocal imaging revealed that the early cone cell marker Visinin could be detected in PH3^+ ^cells (Figure 6E–H), suggesting that the onset of Visinin expression begins during the M phase. This was further confirmed in dissociated retinal cell cultures, where we detected co-labeling of PH3 with weak Visinin signals in anaphase progenitors (Figure 6I–L). Furthermore, confocal imaging demonstrated that intensely Visinin^+ ^cone photoreceptor precursors expressed no Pax6, whereas cells emerging from the telophase expressed both low levels of Pax6 and Visinin (Figure 6M–P). These data suggested that the emergence of the cone marker Visinin correlated with the down-regulation of Pax6 in a subset of M phase cells. Therefore, the divergence of Pax6 expression among a subset of progenitors in G2/M phase of the cell cycle may reflect neuronal fate specification.

### Effects of altering Pax6 levels on proliferation, neuronal fate specification, and cell death

**Figure 7:**
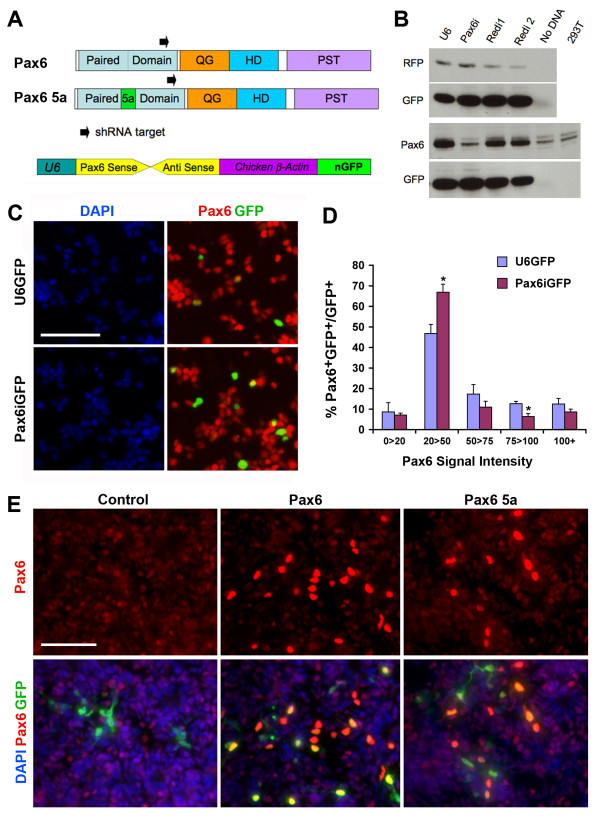
**Effectiveness of Pax6 knockdown and overexpression**. **(A) **Schematics show small hairpin RNA (shRNA) target location in Pax6 and Pax6 5a and the structure of the U6 shRNA construct (bottom). **(B) **Western blots show Pax6 shRNA knockdown in transfected 293T cells. DNAs used are listed on the top and antibodies used are on the left of the blots. U6, control vector used to express shRNA; *Pax6i*, U6 vector expressing shRNA targeting *Pax6*; *Redi1 *and *Redi2*, U6 vectors expressing shRNAs targeting red fluorescent protein (RFP). **(C) **Immunolabeling shows Pax6 protein knockdown in transfected E4.5 (HH stage 24) retinal cells after 2 days *in vitro *(DIV). *Pax6i *transfected samples showed fewer cells co-expressing green fluorescent protein (GFP) and Pax6. **(D) **Quantification of Pax6 protein signal intensities among U6 vector and U6-Pax6i vector transfected E4.5 retinal cells after 2 DIV. Asterisks represent *P*-values between the control and the *Pax6i *samples: *P < 0.05 (N ≥ 4, mean ± standard error). **(E) **Immunofluorescent staining of E5 (HH stage 27) retinal cells transfected with CAG control vector and *Pax6 *or *Pax6*(*5a*) overexpression vectors after 2 DIV. Note both *Pax6 *and *Pax6*(*5a*) expression vector-transfected cells show higher Pax6 expression levels than endogenous Pax6 levels observed in retinal cells.

In order to determine whether Pax6 expression levels are critical in the neurogenic progenitor cells, we perturbed Pax6 expression using small hairpin RNA (shRNA) knockdown and overexpression in the E4 to E4.5 (HH stages 22 to 24) central retina. A conserved 21-nucleotide region in the paired domain shared between *Pax6 *and *Pax6*(*5a*) was selected and cloned downstream of the mouse U6 promoter as a *Pax6 *shRNA cassette (Figure 7A). In transient transfection assays in 293T cells, the shRNA targeting *Pax6 *(*Pax6i*) showed more than 90% efficiency in knocking down co-expressed Pax6 protein, but did not affect expression of the control red fluorescent protein (RFP; Figure 7B). Similarly, E4 (HH stages 23 to 24) primary retinal cells transfected with *Pax6i *showed reduced expression of endogenous Pax6 protein (Figure 7C). This was confirmed by quantification, as the *Pax6i*-transfected population displayed a shift from higher to lower Pax6 levels (Figure 7D). In addition, in retinal cells transfected with vectors overexpressing *Pax6 *or *Pax6*(*5a*) cDNA, significantly higher levels of Pax6 protein compared to endogenous levels were detected (Figure 7E).

**Figure 8:**
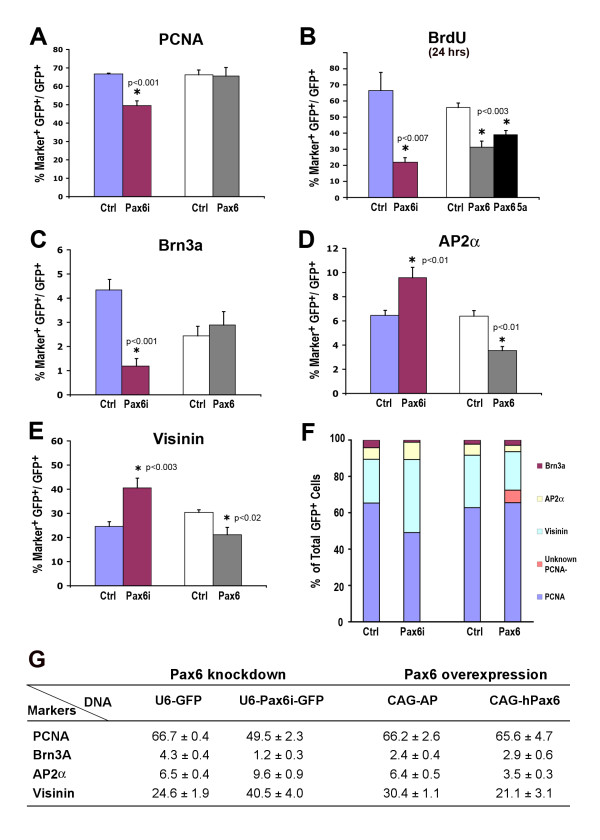
**Effects of altering Pax6 expression on cell proliferation and fate specification**. Chicken retinal explants were transfected with *Pax6 *shRNA expression or *Pax6 *overexpression vectors at E4.5 (HH stage 24). Cell marker assays were performed after 3 days *in vitro*. Statistically significant sample pairs are indicated with asterisks and specific *P*-values (N ≥ 4). **(A-E) **Bar graphs showing percentages of cell marker and green fluorescent protein (GFP) double positive cells over total GFP^+ ^transfected cells. **(F) **Compilation of cell type markers among all transfected GFP^+ ^cells under control and Pax6 perturbation conditions. The mean values were used to generate the graph. **(G) **Summary of the percentages of cell markers and deviations (N ≥ 4, mean ± standard error) under controls and *Pax6 *knockdown or *Pax6 *overexpression conditions. BrdU, bromodeoxyuridine; PCNA, proliferating cell nuclear antigen.

We next examined the effects of altering Pax6 levels during chicken retinogenesis using explant cultures. Quantification of proliferating cell marker PCNA showed that retinas transfected with *Pax6i *at E4.5 (HH stage 24) reduced cycling cells from 66.7% to 49.5%, whereas overexpressing Pax6 did not affect the proportion of PCNA^+ ^cells (Figure 8A,G). In comparison, both *Pax6 *shRNA knockdown and Pax6 overexpression caused significant decreases in overall BrdU incorporation (Figure 8B), suggesting that Pax6 levels affected the proportion of cells undergoing DNA synthesis.

We also examined the influence of Pax6 levels on three major neuronal cell types generated between HH stage 24 and stage 32. Reducing Pax6 by shRNA resulted in a decrease of Brn3a^+ ^retinal ganglion cells from 4.3% to 1.2% (Figure 8C,G). However, Pax6 overexpression did not elevate the production of Brn3a^+ ^RGCs (Figure 8C). Quantification using Visinin showed that reducing Pax6 using shRNA dramatically enhanced cone precursors from 24.6% to 40.5% (Figure 8E,G). Conversely, overexpressing Pax6 caused reduced Visinin^+ ^cone precursor production (Figure 8E). The production of AP2α^+ ^amacrine cells was enhanced when Pax6 was down-regulated and suppressed when Pax6 was elevated (Figure 8D), suggesting that amacrine interneuron specification was also sensitive to Pax6 levels. Together, these results demonstrate that Pax6 expression levels among neurogenic progenitor cells critically affect both cell proliferation and neuronal fate decisions (Figure 8F,G).

**Figure 9:**
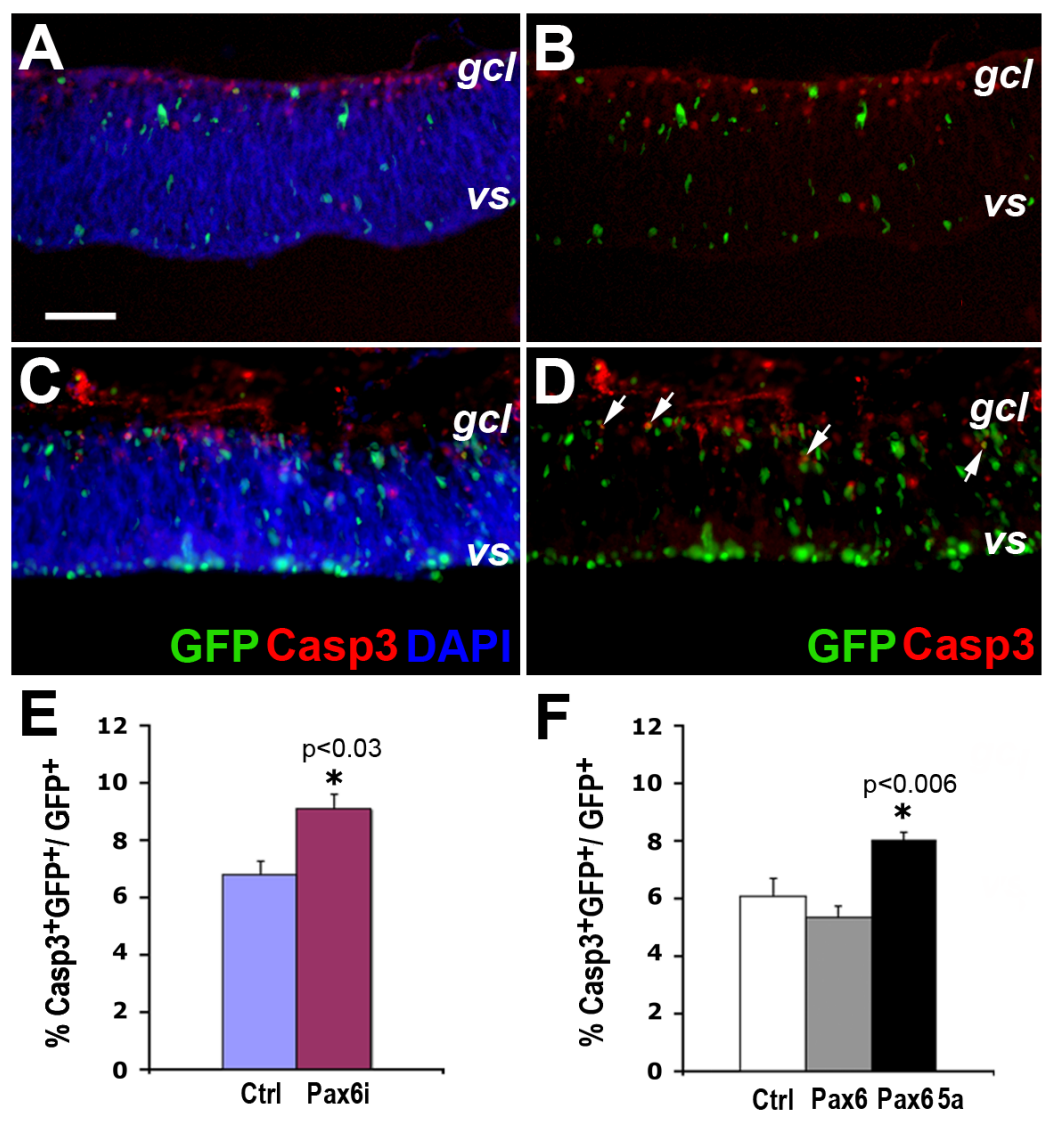
**Effects of altering Pax6 expression on cell death**. **(A-D) **Immunofluorescent images of retinal explants transfected at E4.5 (HH stage 24) with U6 control (A, B) and U6-*Pax6i *(C, D) vectors after 2 days *in vitro*. (A, C) Merged images for green fluorescent protein (GFP), activated caspase 3 (Casp3, red), and DAPI. (B, D) Merged images showing GFP^+ ^transfected cells and Casp3^+ ^signals. Note that *Pax6i*-transfected retinas show higher levels of apoptosis than the U6 control. Arrows in (D) indicate cells co-labeled for GFP and Casp3. **(E, F) **Quantification of Casp3^+ ^cells among GFP^+^-transfected cells in dissociated E5 (HH stage 27) retinal cell cultures after 2 days *in vitro*. Statistically significant data points between the controls and given samples are indicated with asterisks and specific *P*-values (N = 3, mean ± standard error). *gcl*, ganglion cell layer; *vs*, ventricular surface.

Since the small eye phenotype associated with Pax6 mutations may potentially be caused by increased cell death in the retina, we also examined the effects of altering Pax6 expression levels on cell death. In control retinal explants, activated caspase 3 signals were detected; however, they were not often associated with green fluorescent protein (GFP)-positive transfected cells (Figure 9A,B). In contrast, in retinal explants transfected with the *Pax6i *plasmid, increased caspase 3 signals were detected and they often corresponded to GFP^+ ^transfectants (Figure 9C,D). The effects of altering Pax6 expression levels on cell death were further assessed in monolayer retinal cultures. Compared to mock transfected samples, *Pax6i *knockdown increased caspase 3^+ ^cells from 6.8% to 9.1% (Figure 9E). Overexpression of Pax6(5a) but not Pax6 resulted in increased caspase 3^+ ^cells compared to controls (Figure 9F). Therefore, decreasing Pax6 and/or Pax6(5a) or increasing Pax6(5a) resulted in more cell death.

## Discussion

In this study, we have analyzed Pax6 protein expression during the preneurogenic to neurogenic transition and among neurogenic chicken retinal progenitor cells. We provide evidence that Pax6 protein levels are highly dynamic in proliferating progenitors and are coupled with cell cycle progression. We also demonstrate that Pax6 levels critically influence cell proliferation and cell fate specification of neurogenic progenitors.

Pax6 is expressed by retinal progenitors throughout development. However, our results show that Pax6 protein levels are significantly down-regulated as retinal progenitors undergo the preneurogenic to neurogenic transition and initiate production of postmitotic neurons. During the remainder of retinogenesis, the peripheral margin progenitor cells retain a higher level of Pax6 compared to the central progenitor cells. How the down-regulation of Pax6 expression is achieved during the preneurogenic to neurogenic transition remains unknown, but it may involve signals that promote cell-intrinsic changes necessary to enable cell cycle exit and neuronal fate specification. The differing levels of Pax6 in the peripheral versus central retina are achieved, in part, at the transcriptional level through the complex arrays of Pax6 enhancers [17,19]. Our data suggest that translational and/or post-translational regulation may also contribute to the rapid change of Pax6 protein levels during the cell cycle. The cellular distinctions between the central Pax6^Lo ^and peripheral Pax6^Hi ^progenitors are not well understood. Conditional ablations of the *Pax6 *gene in the peripheral retina by Pax6 α-cre and in the central retina by the Chx10-cre both block expression of proneural genes and limit the pluripotency of retinal progenitors [40,41]. However, the peripherally expressed Pax6 also plays a role in suppressing the *Crx *gene [41], suggesting either a context-dependent or dosage-dependent mechanism. The Pax6 perturbation carried out in our study focused on the central neurogenic retina, where the expression of several proneural genes, including *Ath5 *and *Ngn2*, has begun and postmitotic neurons have accumulated. Thus, our experiments address the importance of Pax6 regulation among neurogenic progenitors.

We have established by quantitative flow cytometry analysis that the cycling neurogenic progenitor population expresses a relatively low level of Pax6, which when perturbed interferes with BrdU incorporation. Interestingly, overexpression of Pax6 resulted in a decreased number of cells undergoing DNA synthesis, but did not significantly impact the proportion of PCNA^+ ^cells remaining in the cell cycle. Since PCNA labels proliferating cells in all phases of the cell cycle, this result suggests that high Pax6 levels potentially alter cell cycle distribution and/or lead to cell cycle arrest. Consistent with our observations, studies in the developing cortex have shown that reducing Pax6 perturbs cell cycle length and affects the preneurogenic to neurogenic transition [30,31]. In addition, activation of Pax6 or Pax6(5a) above endogenous levels in the cortical progenitors disrupts cell cycle progression [28]. Live imaging of a tagged Pax6 isoform has revealed that Pax6p46 is associated with the pericentromeric area of the chromosomes during M phase, and overexpression of this isoform causes abnormal mitosis [44]. Our study shows that low levels of Pax6 are expressed by the majority of neurogenic progenitors, but progenitor cells residing within the S phase appear to maintain a more uniformly low level of Pax6 compared to G1 and G2/M phase cells. This raises the possibility that the G1 to S phase transition of neurogenic progenitors may require a narrow range of Pax6 protein. Further analyses are necessary to determine the precise cell cycle defects of retinal progenitors with aberrant levels of Pax6 expression.

Previous findings show that heterozygous Pax6 loss of function mutations lead to microphthalmia in mice [8-12], which is primarily due to defects in early eye morphogenesis. In transgenic mice carrying multiple copies of Pax6, the eye morphology and retinogenesis are initially normal, but RGC axons show dose-dependent projection defects accompanied by postnatal cell loss [12,13]. This suggests that Pax6 levels affect RGC differentiation and survival. Since Pax6 positively regulates transcription of the proneural gene *Math5 *required for RGC development [45], high levels of Pax6 may cause inappropriate Math5 expression, leading to RGC death. Consistent with the finding that aberrant Pax6 levels cause increased apoptosis during cortical development [28], we have detected increased cell death due to reduced Pax6 or increased Pax6(5a) levels in the neurogenic retina. These findings indicate that stringent regulation of Pax6 levels is critical for cell survival at different stages of eye/retinal development.

We have demonstrated that various postmitotic retinal neurons exhibit distinct Pax6 levels, suggesting that Pax6 expression is readjusted when neurogenic progenitors become postmitotic, and that Pax6 may, therefore, function in cell fate determination of progenitors and/or subsequent neuronal differentiation. Our data show that perturbing Pax6 during chicken retinogenesis impacts cell fate specification. In close correlation with the contrasting levels of Pax6 in mature RGCs and cone photoreceptors, decreasing Pax6 led to reduced RGC and enhanced photoreceptor genesis. Conversely, forced expression of Pax6 suppressed photoreceptor production. However, we did not detect increased RGC production due to forced Pax6 expression as previously observed in a low density monolayer chicken retinal culture [46]. This might be due to the progenitors in our retinal explants already expressing sufficient amounts of Pax6 to facilitate RGC fate specification. Alternatively, Pax6 overexpression enhances early neuronal markers but not the more mature RGC marker Brn3a. The majority of postmitotic amacrine cells express high levels of Pax6 in the chicken retina. Our results show that both reducing and elevating Pax6 altered amacrine production, supporting a requirement for Pax6 in either specification or differentiation of amacrine interneurons. This result is consistent with phenotypes of Pax6 conditional knockout in the more centrally localized progenitors, which also lose their pluripotency [41]. The fact that both gain and loss of Pax6 function impede amacrine cell development in the chicken retina suggests that Pax6 is likely required for amacrine cell fate specification rather than differentiation, but direct perturbation of Pax6 in postmitotic amacrine cells is necessary to distinguish these possibilities.

In this study, we present the first analysis of Pax6 protein dynamics with regard to the neurogenic cell cycle. We provide evidence that a subset of neurogenic progenitor cells begins to display divergent Pax6 protein levels upon entering into the G2 phase of the cell cycle. This cell cycle-correlated Pax6 protein level change is more readily detectable in the later neurogenic retina (Figures 4, 5, and 6). The dramatic changes of Pax6 levels among the subset of progenitors, coupled with the relatively short G2 phase, indicate that Pax6 protein in neurogenic progenitors may be subjected to rapid proteolytic degradation. In addition, the correlation between decreased Pax6 expression and the appearance of the neuronal marker Visinin in late M phase cells suggests that the subset of progenitor cells showing divergent Pax6 expression in G2/M are likely those that have committed to certain cell fates. Therefore, neuronal fate determination is likely to have occurred prior to the G2 phase of the neurogenic cell cycle, which produces at least one postmitotic neuron.

**Figure 10:**
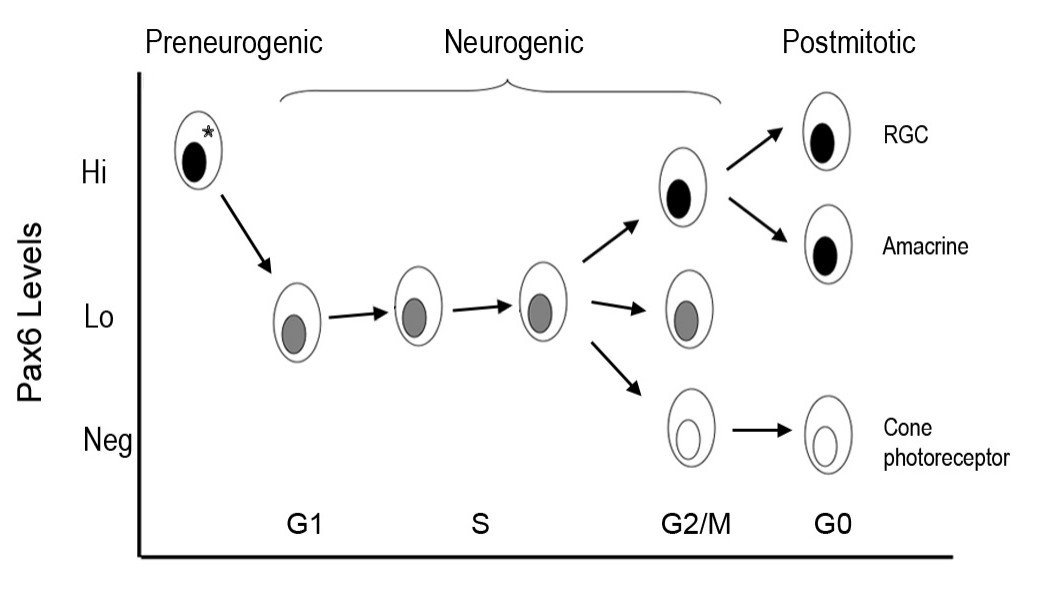
**Pax6 expression during cell cycle progression and a model of cell cycle-dependent Pax6 regulation**. A model of dynamic Pax6 expression during retinogenesis is depicted. Pax6 protein levels decrease in progenitor cells during the transition from the preneurogenic (indicated with an asterisk) to the neurogenic stage. Neurogenic progenitors express low levels of Pax6 protein, which is essential for maintaining the proliferative state, especially the reentry of S phase. The cohort of S phase cells contains a relatively uniform level of low Pax6. Upon entering into the G2/M phase, the neurogenic progenitor population displays more divergent Pax6 levels, with a subset of progenitor cells showing increased or decreased Pax6 protein expression depending on the developmental stage. Because postmitotic neurons contain distinct levels of Pax6 protein, the divergence of Pax6 expression during the G2/M phase may forecast cell fate specification at or prior to the G2 phase of the cell cycle. Postmitotic neurons achieve and maintain specific and distinct levels of Pax6 during differentiation. RGC, retinal ganglion cell.

Based on our results, we propose that preneurogenic retinal progenitor cells maintain a high level of Pax6 (*Pax6^Hi^), which by an unknown mechanism undergoes a substantial down-regulation when cells enter the neurogenic phase. Neurogenic progenitors maintain a low level of Pax6 (Pax6^Lo^), which is essential for sustaining cell proliferation, survival, and neuronal fate selection. Under the influence of unidentified extrinsic and/or intrinsic cues, a subset of progenitors alters their Pax6 levels upon entry into the G2/M phase in preparation for cell cycle exit. Postmitotic neurons display distinct cell type-specific levels of Pax6, ranging from high (Pax6^Hi^) to no Pax6 (Pax6^Neg^), whereas G1 progenitor cells maintain or regain the characteristic low level of Pax6 (Pax6^Lo^) before the G1 to S transition (Figure 10). Results reported here reveal important functions of Pax6 during the neurogenic phase of retinal development and the dynamic nature of Pax6 protein regulation within the cell cycle. Future investigations probing mechanisms that regulate this cell cycle-dependent expression of Pax6 among progenitor cells will enhance our understanding of how progenitor proliferation and fate specification are coordinated.

## Conclusion

During the preneurogenic to neurogenic transition, retinal progenitor cells undergo dramatic down-regulation of Pax6 protein. A low level of Pax6 protein is essential for S phase entry and normal proliferation of neurogenic progenitors. A subset of neurogenic progenitors rapidly increase or decrease Pax6 levels in the G2/M phase of the cell cycle, forecasting cell fate selections prior to G2. The dynamic regulation of Pax6 protein within the cell cycle is critical for the expansion and fate specification of neurogenic progenitors.

## Materials and methods

### Chicken embryos

Fertilized White Leghorn chicken eggs were purchased from Charles River (Wilmington, MA, USA) and were incubated at 38°C in a rotating humidified incubator. Embryos were staged (HH stage) according to Hamburger and Hamilton [47].

### DNA constructs

Overexpression constructs were generated by cloning the human *Pax6 *and *Pax6*(*5a*) cDNAs downstream of the chicken beta actin enhanced cytomegalovirus promoter (CAG)[48] followed by an internal ribosomal entry site upstream of the human placental alkaline phosphatase. For shRNA-mediated knockdown, hairpin sequences specifically targeting *Pax6 *or the control RFP message (HcRed N1; Clonetech, Mountain View, CA, USA) were cloned downstream of the mouse U6 promoter in the vector pBS/U6 (U6) [49]. In addition, a cassette containing the CAG promoter and a nuclear GFP was cloned downstream of the of U6 shRNA cassette in the same orientation. The set of specific primers used to generate the shRNAs targeting Pax6 were: XJY276 (5'GGTCTGTACCAACGATAACAA); XJY277 (5'AGCTTTGTTATCGTTGGTACAGACC); XJY278 (5'AGCTTTGTTATCGTTGGTACAGACCCTTTTTG); XJY279 (5'AATTCAAAAAGGGTCTGTACCAACGATAACAA). The two sets of specific primers used to generate the shRNAs *Redi1 *and *Redi2 *targeting HcRed N1 (Clonetech) were:

Redi1, XJY694 (5'GGCACCGTGAACGGCCACTACTTA), XJY695 (5'AGCTTAAGTAGTGGCCGTTCACGGTGCC), XJY696 (5'AGCTTAAGTAGTGGCCGTTCACGGTGCCCTTTTTG), XJY697 (5'AATTCAAAAAGGGCACCGTGAACGGCCACTACTTA); Redi2, XJY698 (5'GGAGAGAACCACCACCTACGAA), XJY699 (5'AGCTTTCGTAGGTGGTGGTTCTCTCC), XJY700 (5'AGCTTTCGTAGGTGGTGGTTCTCTCCCTTTTTG), XJY701 (5'AATTCAAAAAGGGAGAGAACCACCACCTACGAA).

### Retinal cultures and transfection assays

For transfection of monolayer cells, dissociated HH stage 27 retinal cells were plated on poly-D-lysine coated 8-well LabTek (Nalge Nunc, Rochester NY, USA) slides at 20,000 cells/mm^2^, 3 hours prior to Lipofectamine PLUS (Invitrogen, Carlsbad, CA, USA) transfection using *Pax6 *shRNA knockdown vectors or Pax6 overexpression vectors with a CAG-nGFP plasmid at 2:1 ratio [50]. For transfection of retinal tissues, DNA at 2 μg/ml in phosphate-buffered saline (PBS) was injected into the subretinal space of the eye at HH stage 24/25. The injected eyes were placed between paddle electrodes and subjected to five pulses at 10 V/mm for 25 ms with 975-ms intervals using ECM 830 Square Wave Electroporation System (BTX, Holliston, MA, USA). Central retinas (posterior halves) were dissected from electroporated eyes and cultured on Nucleopore membrane discs (Millipore, Billerica, MA, USA) floating on a retinal culture medium containing an equal ratio of F12:DMEM (JRH Biosciences, Lenexa, KS, USA), 2% fetal calf serum, 0.2% chicken serum, 10 mM HEPES, and penicillin/streptomycin at 37°C in 5% CO_2 _for 72 hours.

Transfection of 293T cells was carried out using Lipofectamine PLUS (Invitrogen) and 0.65 μg of U6-Pax6i, U6-Redi1, U6-Redi2, or U6-GFP with 0.5 μg of either RCAS(B)-hPax6 DNA or CMV-HcRedN1 (RFP) as well as 0.35 μg of CAG-nGFP as transfection control. At 48 hours post-transfection, cells were lysed in RIPA buffer without deoxycholate, protein extracts were resolved on a 10% BisTris SDS-PAGE gel using MOPS buffer (Invitrogen), and transferred onto nitrocellulose membrane. Blots were probed with antibodies against GFP (1:500; Invitrogen), HcRedN1 (1:500; Becton Dickenson, Laguna Hills, CA, USA), Pax6 (1:1,000; Chemicon/Millipore, Billerica, MA, USA), and followed by secondary antibodies conjugated with horseradish peroxidase and detected by enhanced chemiluminescence (ECL plus; Amersham, Piscataway, NJ, USA).

### Immunohistochemistry and imaging

Heads of chicken embryos or retinal explants were fixed in 4% paraformaldehyde in PBS overnight at 4°C, followed by cryoprotection with 30% sucrose in PBS and embedding in OCT. Coronal sections 20 μm thick were collected and used for immunolabeling. For BrdU pulse labeling, the posterior half of HH stage 30 or stage 33 to 34 retinas were dissected and incubated in the retinal culture medium supplemented with 20 μm BrdU for 20 minutes, followed by extensive washes in fresh culture medium without BrdU, and then further cultured as free floating explants for designated time periods. Retinal cryosections labeled with BrdU were treated with 95% formamide in 1× standard sodium citrate (SSC) pH7.4 for 10 minutes at 70°C prior to antibody incubation.

Immunohistochemistry was performed as previously described [51]. Cryosections or monolayer cells were incubated with the following primary antibodies: Neurofilament 68 (mouse, 1:200; Sigma Aldrich, St. Louis, MO, USA), Neurofilament 145 (rabbit, 1:500; Chemicon), Phospho-histone H3 ser28 (rabbit, 1:500; Millipore), Phospho-histone H3 ser10 (rat monoclonal, 1:250; Sigma), Pax6 (mouse, 1:10; Developmental Study Hybridoma Bank, Iowa City, IA, USA), Pax6 (rabbit, 1:500; Chemicon and Covance, Princeton, NJ, USA), PCNA (mouse, 1:200; Sigma), Brn3a (mouse, 1:200; Chemicon), AP2α (mouse, 1:5; DSHB), Islet 1/2 (mouse clone 39.4D5, 1:10; DSHB), Visinin (mouse, 1:50; DSHB), activated caspase 3 (rabbit, 1:250; Upstate/Millipore), BrdU (mouse, 1:5; Amersham), and GFP (mouse monoclonal and rabbit polyclonal; Invitrogen). Sections were then incubated with either biotinylated secondary antibodies followed by horseradish peroxidase detection (Vector Laboratories, Burlingame, CA, USA) using 3,3'-diaminobenzidine (Sigma), or appropriate secondary antibodies conjugated to Alexa 488, 568, 594, or 647 (Invitrogen). For nuclear staining, 1 mg/ml of 4',6-diamidino-2-phenylindole (DAPI; Roche, Palo Alto, CA, USA) was used. Epifluorescent and DIC images were captured using a Nikon E800 microscope equipped with a SPOTII digital camera. Confocal optic sections 1 μm thick were obtained using a Zeiss LSM 410 confocal laser-scanning system with a Zeiss Axiovert 135M microscope, or a Leica TCS SP MP confocal microscope.

### Flow cytometry

The anterior (st25 analysis) and posterior halves of retinas were dissected and collected in Hank's buffer saline solution (HBSS^+^; Invitrogen). Retinas were washed in HBSS without Mg^+ ^and Ca^+ ^(HBSS^-^; Invitrogen) and incubated with 0.1% trypsin (Sigma) at 37°C for 10 minutes. Soybean trypsin inhibitor (Sigma) at a final concentration of 0.1 mg/ml or chicken serum (Sigma) to 10% of total volume was added to stop trypsin, followed by addition of DNaseI (Sigma) to 50 mg/ml and trituration to obtain single cell suspensions. Single cell suspensions were fixed in 2% paraformaldehyde in PBS for 30 minutes, washed with HBSS^+^, followed by 0.1% Triton-X in HBSS^+ ^incubation prior to staining with primary and secondary antibodies as described above with washes in between. Anti-PCNA and anti-BrdU antibody labeled cells were also treated with 2 N and 1 N HCl, respectively, prior to antibody labeling. Cells were filtered and collected using a tube with a 35 mm nylon mesh cap (BD Biosciences/Falcon, San Jose, CA, USA) before flow cytometry analyses using an LSR flow cytometer (BD Biosciences).

### Quantification of cell markers and statistical analysis

Quantification of various cell markers for dissociated retinal cells was performed by counting non-overlapping fields through the microscope directly or by analyzing acquired digital images using ImagePro PLUS software (Media Cybernetics, Bethesda, MD, USA). For each sample, a minimum of 200 marker-positive cells or the entire 7-mm diameter well was counted. Quantification of dissociated retinal cells by FACS was performed using FlowJo v5.7.2 according to the manufacturer's guidelines (Ashland, OR, USA). For each sample, a minimum of 30,000 cells was analyzed. Student *t*-test was applied to data sets where *P *< 0.05 is considered statistically significant. Data were presented as mean ± SEM.

## Abbreviations

BrdU: bromodeoxyuridine; CMV: cytomegalovirus; E: embryonic day; GFP: green fluorescent protein; HH: Hamburger and Hamilton; PBS: phosphate-buffered saline; PCNA: proliferating cell nuclear antigen; PH3: phospho-histone 3; RFP: red fluorescent protein; RGC: retinal ganglion cell; shRNA: small hairpin RNA.

## Competing interests

The authors declare that they have no competing interests.

## Authors' contributions

YWH participated in the design of the study, carried out the experiments, and helped to draft the manuscript. X-JY participated in the design of the study and wrote the manuscript. Both authors read and approved the final manuscript.

## Supplementary Material

Additional file 1 **Pax6 levels among cells in different phases of the cell cycle**. Pax6 levels among cells in different phases of the cell cycle. Click here for file
