# Supplementary material for: Dynamic Pax6 expression during the neurogenic cell cycle influences proliferation and cell fate choices of retinal progenitors
Source: Neural Dev. 2009 Aug 17;4:32. doi: 10.1186/1749-8104-4-32 (PMC2741438; doi:10.1186/1749-8104-4-32)
Supplement: Additional file 1 — Pax6 levels among cells in different phases of the cell cycle. Pax6 levels among cells in different phases of the cell cycle. [file 1749-8104-4-32-S1.doc]

Suppl. Table 1.

Pax6 Levels among Cells in Different Phases of the Cell Cycle

**G0/G1** **S**  **G2/M**

DNA Content (2n) (>2n, <4n) (4n)

**Pax6 Level**

Pax6^Hi^ 9.9 ± 0.7 4.1 ± 0.6 9.1 ± 0.5

Pax6^Lo^ 71.2 ± 0.4 90.0 ± 0.5 87.2 ± 0.5

Pax6^Neg^ 18.8 ± 0.6 5.8 ± 0.3 3.8 ± 0.2

**Percent of Total Cells**

73.1 ± 1.0 8.7 ± 0.4 18.1 ± 0.8

* Dissociated HH stage 30 central retinal cells (N=6) were subjected to flow cytometric analysis for their Pax6 levels and DNA contents.

**Percentages of cells with different Pax6 levels are calculated within each DNA content category.
